# A Phase Ib Study of Indirect Immunization with Oregovomab and Toll-like-Receptor-3 Stimulation with Hiltonol^®^ in Patients with Recurrent Platinum-Resistant Ovarian Cancer

**DOI:** 10.3390/curroncol32100532

**Published:** 2025-09-24

**Authors:** Robert W. Holloway, Sarah M. Temkin, Sarah W. Gordon, Sunil Gupta, Srinivasa R. Jada, Sarfraz Ahmad, William P. McGuire

**Affiliations:** 1 Gynecologic Oncology Program, AdventHealth Cancer Institute, Orlando, FL 32804, USA; 2 Division of Gynecologic Oncology, Virginia Commonwealth University, Richmond, VA 23298, USA; 3 CanariaBio, Inc., Seoul 06044, Gyeonggi-do, Republic of Korea

**Keywords:** ovarian cancer, platinum-resistance, recurrent disease, oregovomab, Hiltonol^®^, indirect immunization, TLR3 stimulation, phase Ib study, survival analysis, CA-125

## Abstract

*Objectives*: This phase Ib study assessed the safety and compatibility of indirect oregovomab immunization and Toll-like-receptor-3 (TLR3) stimulation with immune adjuvant Hiltonol^®^ (poly-ICLC) and induced clinically relevant CA125-specific anti-tumor immunity in heavily pretreated patients with progressive platinum-resistant ovarian cancer (PROC). *Methods*: Patients with elevated serum CA125 level >50 U/mL received four intravenous infusions with 2 mg oregovomab followed by 2 mg Hiltonol^®^ intramuscular 30 min and 48 h post-oregovomab at weeks 0, 3, 6, and 9. At week 12, imaging was performed, and salvage chemotherapy was allowed post-progression per the investigator’s discretion. The Fifth/final oregovomab with Hiltonol^®^ infusion was given at week 16. *Results*: Fifteen enrolled patients were analyzed for safety and efficacy. Thirteen (87%) patients completed at least three Hiltonol^®^ infusions with oregovomab, specifically, two cycles (*n* = 2), three cycles (*n* = 2), four cycles (*n* = 3), and five cycles (*n* = 8). Adverse events included mild fatigue, flu-like symptoms, chills, axillary pain, and injection site discomfort in 13 (87%) patients. Serious adverse events were reported in seven (47%) patients, including Grade 3 hypertension (*n* = 2), thrombocytopenia (*n* = 1), and Grade 3 events attributed to underlying disease (*n* = 4). Ten (67%) patients had disease progression, three (20%) had stable disease, and two were unevaluable. Early humoral response by week 6 was observed in seven of nine (77%) patients, median progression-free survival was 2.7 months (95% confidence interval [CI]: 2.2, 3.3), and median overall survival was 15.0 months (95% CI: 8.2–23.9). *Conclusions*: The safety and compatibility of combining oregovomab with Hiltonol^®^ have been demonstrated in this study. The potential to enhance activity of chemotherapy using oregovomab indirect immunization and Hiltonol^®^ stimulation is proposed.

## Simple Summary

This phase Ib study assessed the safety and compatibility of indirect oregovomab immunization and Toll-like-receptor-3 stimulation with immune adjuvant Hiltonol^®^ and induced clinically relevant cancer antigen-125-specific anti-tumor immunity in heavily pretreated patients with platinum-resistant ovarian cancer. Patients with elevated serum CA125 level > 50 U/mL received four intravenous infusions with 2 mg oregovomab followed by 2 mg Hiltonol^®^ intramuscular 30 min and 48 h post-oregovomab at weeks 0, 3, 6, and 9. At week 12, imaging was performed, and salvage chemotherapy was allowed post-progression per the investigator’s discretion. Fifteen enrolled patients were analyzed for safety and efficacy. Thirteen patients completed at least three Hiltonol^®^ infusions with oregovomab. Adverse events included mild fatigue, flu-like symptoms, chills, axillary pain, and injection site discomfort. Treatment-related serious adverse events included hypertension (*n* = 2) and low platelets (*n* = 1). Median progression-free survival and overall survival were 2.7 and 15 months, respectively. This study demonstrated safety and compatibility.

## 1. Introduction

Ovarian cancer treatment remains a challenge, with most patients having advanced-stage disease at diagnosis. Despite improvements in survival through advances in surgery, chemotherapy, and maintenance strategies, overall cure rates have only modestly improved in recent decades. Although the initial response to cytotoxic therapies is usually encouraging, most patients develop a recurrence of disease that eventually becomes “platinum resistant”, defined by progression-free survival of less than six months, including “platinum refractory” patients who progress on or within one month of platinum doublet therapy. Platinum-resistant ovarian cancer (PROC) represents a significant unmet medical need, and once platinum resistance develops, median survival is approximately one year. Significant improvements in survival for cervical and endometrial cancers with the use of immune checkpoint inhibitors have been demonstrated in recent years, yet their utility in ovarian cancer has been disappointing. Epithelial ovarian cancer is considered immunologically “cold”, and checkpoint inhibition has had modest clinical efficacy. Therefore, innovative approaches are necessary to improve outcomes for patients with advanced epithelial ovarian cancer.

Indirect immunization can produce anti-tumor immunity in patients with ovarian cancer through the administration of low-dose tumor antigen-specific antibody to patients in a limited number of infusions. Oregovomab (MAb B43.13) is a cancer antigen (CA)-125-specific high-affinity murine monoclonal antibody (IgG1) that has been extensively evaluated in clinical trials, primarily in patients with ovarian cancer expressing CA-125 [1,2,3,4,5,6]. Oregovomab has been shown to activate both humoral and cellular immune responses through enhanced antigen uptake and cross-presentation to T-cells in ovarian cancer [7].

Phase I and II studies with oregovomab have demonstrated clinical activity and/or more potent bioactivity of oregovomab in combination with platinum doublet therapy for primary and recurrent disease, respectively [1,3,8], despite the presumed immunosuppressive state of these patients. However, efforts to avoid the immune suppression associated with tumor burden and treatment with cytotoxic agents failed to demonstrate clinical activity when used in the maintenance setting [2]. Oregovomab is currently in phase III evaluation of front-line chemoimmunotherapy (NCT04498117), having shown benefits with combination with chemotherapy relative to chemotherapy alone in a randomized phase 2 study [4].

Hiltonol^®^ (Oncovir, Inc., Washington, DC, USA), or polyinosinic–polycytidilic acid (poly-ICLC), is a Toll-like receptor-3 (TLR3) agonist that has been extensively studied as a cancer immune adjuvant using a variety of tumor vaccination strategies [9,10,11] but has not previously been combined with oregovomab or other indirect immunizing antibodies. Hiltonol^®^ is a synthetic double-stranded RNA (dsRNA) viral mimic and host-defense activator. The proposed mechanism of action is a tumor pro-apoptotic effect that induces tumor neoantigen expression and promotes the induction of natural killer (NK) cell activity. Hiltonol^®^ acts as a critical immune adjuvant for cancer vaccines, whose mechanisms include TLR3 and melanoma differentiation-associated gene/protein 5 (MDA5)-mediated systems. Hiltonol^®^ induces killer-cell targeting (chemotaxis) and tumor vascular endothelial infiltration and supports the host’s ability to overcome immune suppression “checkpoints” in the tumor microenvironment (TME) via a cluster of differentiation 8 (CD8) boost with anti-programmed cell death-1 (aPD-1) checkpoint blockers. It also activates immunological memory, thereby facilitating durable tumor responses.

In preclinical studies, TLR3 stimulation has been shown to enhance antigen processing and further stimulate the specific immunity achievable with antibody and antigen immunization [12]. The addition of the TLR3 danger signal in association with this immunization is expected to magnify the immune response induced in patients with cancer. The dosing phase of this study established the safety and compatibility of this combination (i.e., oregovomab and Hiltonol^®^), and the primary safety and response outcomes are presented. Herein, we report the final clinical and immunological outcomes following interval indirect immunization with oregovomab and Hiltonol^®^ in patients with recurrent PROC. The *primary objective* of this study was to evaluate the safety and compatibility of the combination of oregovomab and Hiltonol^®^ as a strategy to induce CA-125-specific anti-tumor immunity in heavily pretreated patients with progressive ovarian cancer. The *secondary objective* was to evaluate the preliminary activity of the combination of oregovomab and Hiltonol^®,^ including immune response, clinical response, progression-free survival (PFS), and overall survival (OS).

## 2. Materials and Methods

**Figure 1:**
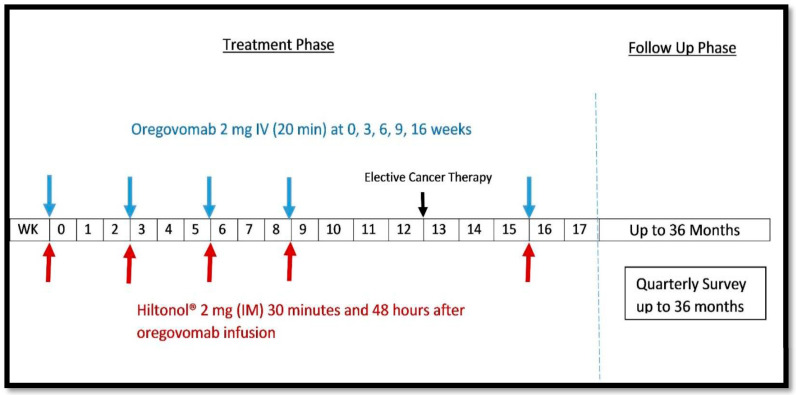
Schematic representation of oregovomab and Hiltonol^®^ treatment and follow-up phases in patients with platinum-resistant ovarian cancer. Abbreviations: IV = Intravenous; WK = Week; IM = Intramuscular.

This was a prospective phase 1b study evaluating the safety and activity of the combination of oregovomab and Hiltonol^®^ in patients with PROC. This study combined oregovomab, in a dose used in prior ovarian cancer indirect immunization protocols (2 mg IV Q3 to 6 weeks, 4 doses), with the currently recommended dose of Hiltonol^®^ (2 mg intramuscular (IM) at 30 min and 48 h post-oregovomab) at weeks 0, 3, 6, and 9. At week 12, imaging was performed, and elective salvage chemotherapy was allowed post-progression as per the investigator’s discretion. A fifth and final infusion of oregovomab with Hiltonol^®^ was to be given at week 16, and patients were followed for up to an additional 3 years to assess recurrence and survival on subsequent palliative therapies (Figure 1). CT scans were performed per protocol at 12-week intervals unless clinical indications required earlier scans.

Patients with PROC who had at least one platinum doublet regimen with progression of disease within six months of the last cycle of therapy and an elevated serum CA-125 level > 50 U/mL measured within 4 weeks of enrollment were considered for enrollment. Patients must have had RECIST (Response Evaluation Criteria in Solid Tumors) lesions and an ECOG performance of 0 to 1. Patients with bowel obstruction, hepato-renal failure, or active brain metastases were excluded. 

Patients were screened for eligibility up to 4 weeks before treatment. Informed consent was obtained before any screening evaluations were completed. All clinical, safety, and immunological evaluations were conducted according to a defined schedule with study termination at week 17. In total, eighteen patients were screened, and three patients did not meet all the inclusion criteria. Fifteen patients with platinum-resistant, high-grade serous carcinoma of the ovary who met the inclusion criteria and received at least one dose of the study drug were included in the intent-to-treat (ITT) population for efficacy and safety analysis. Upon completion of the immunization protocol, all patients were invited to participate in a quarterly outcomes survey for up to 3 years.

### Study Variables and Statistical Analyses

The *primary endpoint* was safety: Treatment-Emergent Adverse events and clinically significant changes in the subjects’ physical examination findings, vital signs, and clinical laboratory results were evaluated. The *secondary endpoints* were as follows: (i) clinical response during 12-week immunization (using Gynecologic Cancer Intergroup (GCIG)/modified immune-related RECIST (irRECIST1.1) criteria), (ii) response to continuing elective combinatorial therapy post week-12, (iii) progression-free survival (PFS), (iv) overall survival (OS), and (v) humoral and cellular immune response. Blood samples were obtained for translational studies, including CA-125-specific T-cell immunity and human anti-mouse antibodies (HAMA) at baseline, 6 weeks (HAMA), and 12 weeks (HAMA plus T-cell immunity). At 17 weeks, the end of the active study period, an additional blood sample was obtained for HAMA and T-cell immunity.

Clinical response was evaluated for each patient using a composite of clinical parameters based on RECIST and iRECIST. PFS was calculated as the time from the first dose of oregovomab to the date of progression of disease, as confirmed by clinical evaluations and RECIST measurements on CT scans, and date of death. Overall survival was calculated using the date of the first dose of oregovomab to the date of death, up to a maximum of 3 years post first treatment. Patients alive at the time of the analysis or who were lost to follow-up were censored at the date they were last known to be alive. For PFS and OS, the analyses consisted of descriptive Kaplan–Meier summaries of median PFS, as well as the 25th and 75th percentiles. A Kaplan–Meier plot of PFS and OS was produced for each parameter. The OS and PFS were summarized overall and by the baseline (neutrophils + monocytes)-to-lymphocytes ratio (NMLR) category, defined as ≤4.0 versus >4.0. 

Tabulations were produced for appropriate demographic, baseline, efficacy, and safety parameters. For categorical variables, summary tabulations of the number and percentage of patients within each category (with a category for missing data) of the parameter are presented. For continuous variables, the number of patients, mean, median, standard deviation (SD), minimum, and maximum values are presented. 

## 3. Results

Patients’ demographics and clinical characteristics are summarized in Table 1. Fifteen patients were enrolled and dosed in two centers. Of the fifteen patients in the ITT population, two were unevaluable, as one patient died prior to the planned week 12 disease assessment and a second withdrew consent for further study treatment and assessments following baseline lesion assessment. Thirteen patients completed the specified minimum three infusions and are included in the efficacy analysis. Median age was 68 years, 80% were White, two-thirds had ECOG performance 0 with remaining performance of 1, and the median number of prior lines of therapy was five (range 3 to 8). Median pre-immunization CA-125 levels were 357 U/mL (range 63 to 2140). The majority of patients (80%) had epithelial ovarian cancer, and the remaining patients had primary peritoneal serous cancer (13.3%) or serous fallopian tube cancer (6.7%). The median time from initial diagnosis was 53.6 (range 25 to 110) months. All patients had at least one recurrence, and the time from last platinum therapy to progression was <1 month for nine (60%) patients and 1 to 6 months for the remaining six (40%) patients, with the median time to recurrence following last platinum therapy of 0.5 months (range 0–11 months) (Table 1).

Thirteen of fifteen (87%) enrolled patients completed at least three doses of oregovomab plus Hiltonol®, and all underwent cytotoxic therapy following week 12 (Table 2). Ten (66.7%) patients had modified iRECIST progression of disease (PD), three (20%) had stable disease (SD) as the best response, and two patients were unevaluable.

**Figure 2:**
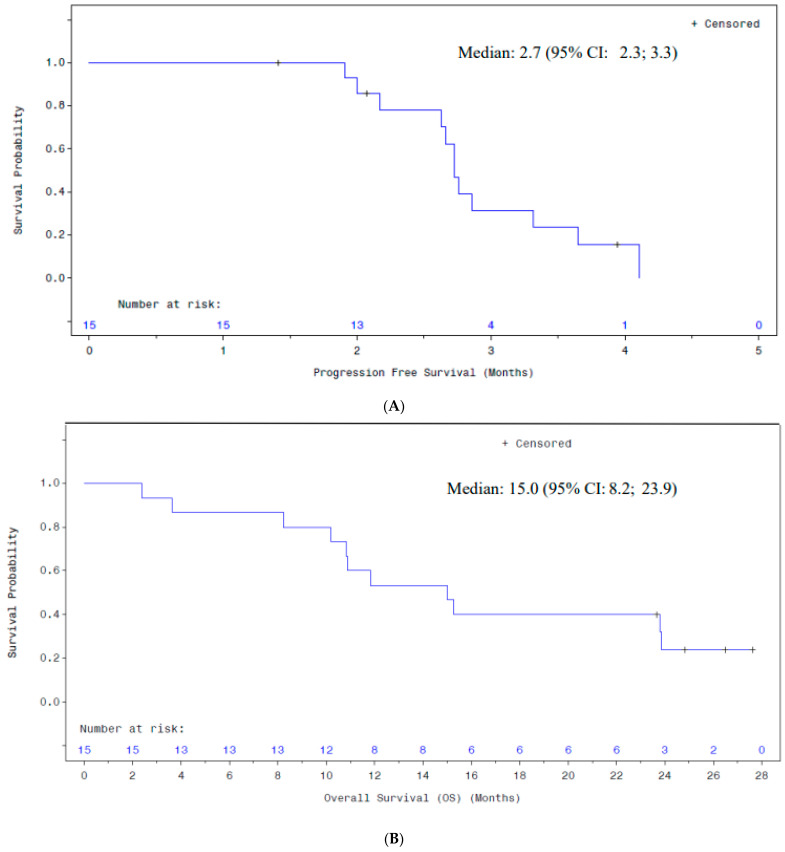

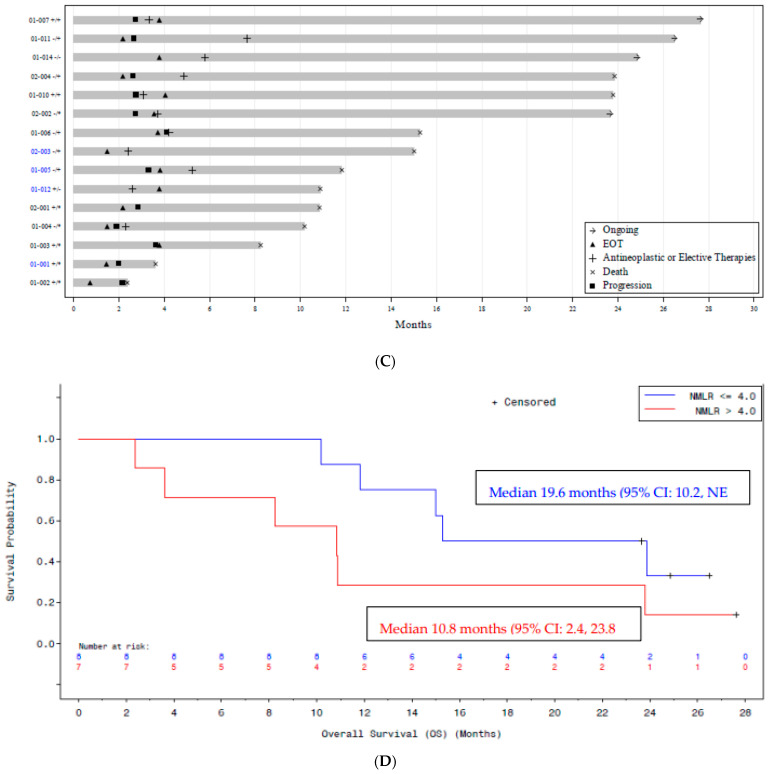

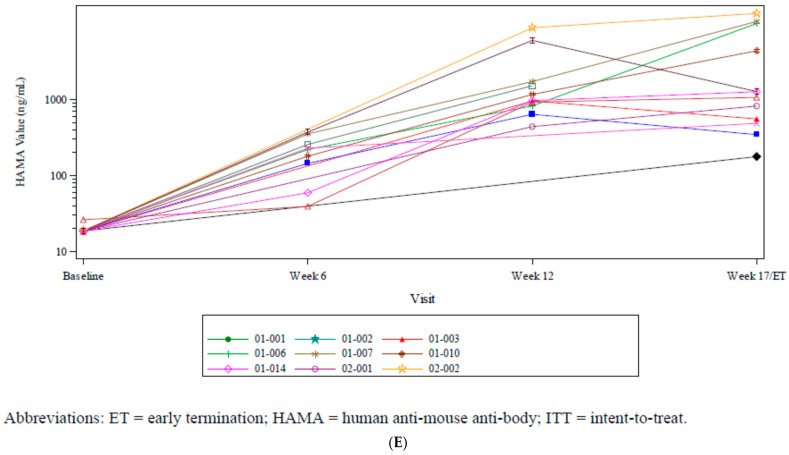
(**A**) Progression-Free Survival: ITT Population; (**B**) Overall Survival: ITT Population; (**C**) Swimmer Plot of Survival (ITT Population); (**D**) Overall Survival by Baseline NMLR (ITT Population; (**E**) Spaghetti Plot of HAMA Values Over Time By Patient (ITT Population).

The median PFS was 2.7 months (95% confidence interval [CI]: 2.2, 3.3). Median survival was 15.0 months (95% CI: 8.2–23.9 months), and four patients remained alive at the time of data lock. Figure 2A,B illustrate the Kaplan–Meier analyses of PFS and OS for the intent-to-treat (ITT) population (*n* = 15). A Swimmer’s plot of OS for all the ITT patients is shown in Figure 2C. Twelve patients (80.0%) experienced disease progression, and eleven (73.3%) died during the study. Eleven patients (73.3%) initiated new antineoplastic therapy, including three patients (20%) who began new cancer therapy before concluding treatment with the study drug and were therefore censored for PFS analysis. Four patients (26.7%) were reported to be alive at the last follow-up contact.

The baseline neutrophil–monocyte-to-lymphocyte ratio (NMLR), a measure of myeloid-derived immune suppression, was inversely associated with survival (Figure 2D). Patients with a baseline NMLR ≤4x (*n* = 8) had a median OS of 19.6 months (CI 10.2 to NE) compared to a median OS of 10.8 months (CI 2.4 to 23.8) for patients with an NMLR >4.0 (*n* = 7). 

Hiltonol^®^ stimulated an early humoral antibody response to oregovomab at week 6 in nine (78%) patients. Interval administration of second-line treatment (bevacizumab, paclitaxel, carboplatin, and/or pegylated-doxorubicin) and oregovomab was associated with further antibody spikes (Figure 2E).

The patients’ safety summary is provided in Table 3. All Treatment-Emergent Adverse Events (TEAEs) were coded using the MedDRA dictionary. Fifteen patients experienced at least one TEAE. The most common TEAEs were fatigue (53.3%), nausea (46.7%), influenza-like illness (40%), and chills (26.7%). Fatigue, local administration site reactions, and mild flu-like symptoms were reported in 13 (86.7%) patients. Serious adverse events were reported in seven (47%) patients, including Grade 3 hypertension in two patients (13%) and Grade 3 thrombocytopenia in one (7%) patient, and the remaining four Grade 3 events were attributed to underlying disease and not considered treatment-related. No dose reduction or discontinuation of the study drug due to AEs occurred during the study, and no AEs resulted in deaths. 

Table 4 summarizes the Grade 3 toxicity associated with the underlying disease. Seven patients (46.7%) experienced at least one Grade ≥3 TEAE. No patients experienced Grade 4 or Grade 5 TEAEs (Listing 16.2.7.1). The only Grade 3 event that occurred in more than one patient was hypertension (two patients, 13.3%). One patient experienced a Grade 3 thrombocytopenia that was assessed as probably related to Hiltonol^®^; all other Grade ≥3 events were considered to be unrelated to the study drugs.

## 4. Discussion

Indirect immunization with tumor-specific antibody is a promising approach to therapeutic immunity against cancer through the activation of immune cells. Hiltonol^®^ is a viable immune adjuvant for combining with oregovomab and is suitable for study in immune-resistant settings such as epithelial ovarian cancer. Therefore, in this study, we sought to establish the feasibility of adding Hiltonol^®^ to oregovomab as an indirect immunization strategy in patients with platinum-resistant/refractory ovarian cancer. The combination was generally well-tolerated, and no unexpected adverse immune treatment safety signals were detected. Immune responsiveness was similar to that observed in prior studies, where oregovomab was administered on the same day in an alternating schedule of carboplatin–paclitaxel in front-line treatment of ovarian cancer [3,7]. Early humoral response to oregovomab was observed in seven of nine (77.8%) patients by week 6, suggesting that Hiltonol^®^ was boosting early immune response to oregovomab infusion with receiving oregovomab, analogous to the same-day schedule of chemo-immunotherapy, as reported by Braly et al. [7].

Despite the presumed immunosuppressive state of ovarian cancer patients, oregovomab in combination with chemotherapy has demonstrated clinical activity and more potent bioactivity for the treatment of patients with primary and recurrent disease in previous studies [1,3,7]. In a phase II study, treatment with oregovomab combined with paclitaxel–carboplatin resulted in significant improvement in both PFS and OS relative to carboplatin–paclitaxel alone [4]. Moreover, preliminary analysis correlates the treatment effect to the baseline neutrophil lymphocyte ratio (NLR) and to induction of CA-125-specific T-cell immunity [3]. Oregovomab is currently in phase III evaluation of front-line chemoimmunotherapy (NCT04498117) to replicate and confirm the results observed in the phase II study [4]. From prior experience, it seems that patterns of immune response to oregovomab in the setting of recurrent ovarian cancer are influenced by concomitant anti-neoplastic therapy [1]. However, clinical outcomes appear sensitive to the myeloid burden (NMLR >4 being adverse), which may be more prevalent in patients with disease resistant to treatment than in chemotherapy-naïve patients, as previously observed in front-line chemo-immunotherapy trials [3].

Cytotoxic chemotherapy may also cause the release of tumor antigens upon cell death and facilitate phagocytosis by dendritic cells (DCs), which is mediated by damage-associated molecules, and augments antigen presentation in T cells. Cell death can also stimulate type 1 interferon (IFN) secretion by tumor cells via Toll-like receptor 3 (TLR3), which leads to production of the chemokine CXCL10, which has been linked to cancer development and progression [13]. Hiltonol^®^ is an investigational TLR3 agonist that has been found to stimulate both cellular and humoral immune responses. Hiltonol^®^ has been tested as a single-agent therapeutic for multiple malignancies, including advanced renal carcinoma and malignant glioma, and demonstrated adequate safety [14,15]. The current study has established the compatibility of adding Hiltonol^®^ to a schedule of indirect immunization with oregovomab. With the limitation of the small number of patients in the study, the observed safety and humoral response needs to be further evaluated in a larger patient population in a prospective comparative study to confirm the added immunotherapy effect to chemotherapy in the platinum-resistant setting.

## 5. Conclusions

Indirect immunization with oregovomab combined with Hiltonol^®^ was shown to be well tolerated, with mild flu-like symptoms in most patients with platinum-resistant/refractory ovarian cancer in this study. Further exploration of chemo-immunotherapy using indirect immunization with oregovomab plus Hiltonol^®^ should be considered.

## Figures and Tables

**Table 1:** Summary of demographics and clinical characteristics of patients with platinum-resistant ovarian cancer.

Characteristics	Subjects (***N*** = 15)
Disposition (of 18 screened)	
Entered the study and dosed	15
Received 3 doses of immunotherapy	13
Age at study entry (year)	
Median	68
Range	42–83
ECOG performance status, *n* (%)	
0	10 (66.7)
1	5 (33.3)
Race, *n* (%)	
Black	1 (6.7)
White	12 (80)
Spanish	1 (6.7)
Indian	1 (6.7)
Ethnicity, *n* (%)	
Non-Hispanic	13 (86.7)
Hispanic	2 (13.3)
Origin of epithelial cancer, *n* (%)	
Ovarian	12 (80)
Peritoneal	2 (13.3)
Tubal	1 (6.7)
Time since the initial diagnosis (months)	
Median	53.6
Min–Max	25–110
FIGO stage *n* (%)	
IIIC	11 (73.3)
IV	4 (26.7)
Pre-immunization CA-125, U/mL	
Median	357
Min–Max	63–2140
Lines of prior therapy	
Median	5
Min–Max	3–8
Time Since Most Recent Relapse (months)	
Median	0.5
Min–Max	0–11
Time from last platinum therapy	
<1 month	9 (60%)
1–6 months	6 (40%)

Abbreviations: FIGO = International Federation of Gynecology and Obstetrics.

**Table 2:** A summary of the study drug exposure in patients with platinum-resistant ovarian cancer.

Parameter	***N*** (%)
Indirect immunizations	15 (100)
Oregovomab	
2	2 (13.3)
3	2 (13.3)
4	3 (20)
5	8 (53.3)
Hiltonol^®^ injections	
4	2 (13.3)
6	2 (13.3)
8	3 (20)
10	8 (53.3)
Continuing antineoplastic therapy	
Started chemotherapy and stopped immunotherapy	3 (20)
Started chemotherapy during immunotherapy	4 (26.6)
Started chemotherapy post-immunotherapy	6 (40)
Went to hospice prior to week 12	2 (13.3)
Went to hospice post week 17	1 (6.7)

**Table 3:** Safety summary (*N* = 15).

Events	*n* (%)
At Least 1 TEAE	15 (100)
Relationship ^a^	
Not Related	14 (93.3)
Unlikely Related	8 (53.3)
Possibly Related	11 (73.3)
Probably Related	6 (40)
Definitely Related	2 (13.3)
Missing	0
CTCAE Grade	
1	15 (100)
2	13 (86.7)
3	7 (46.7)
4	0
5	0
At Least 1 TEAE	14 (93.3)
At Least 1 Grade ≥3 TEAE	7 (46.7)
At Least 1 Serious TEAE	5 (33.3)
At Least 1 TEAE Leading to Study Withdrawal	0

^a^ The relationship of events is not mutually exclusive; Abbreviations: CTCAE = Common Terminology Criteria for Adverse Events; TEAE = Treatment-Emergent Adverse Event.

**Table 4:** Grade 3 toxicity all associated with underlying disease.

MedDRA System Organ Class Preferred Term	All Subjects (***n*** = 15) ***n*** (%)
At least 1 TEAE with CTCAE Grade ≥3	7 (46.7)
Gastrointestinal disorders	3 (20)
Large intestinal obstruction	1 (6.7)
Nausea	1 (6.7)
Small intestinal obstruction	1 (6.7)
Vomiting	1 (6.7)
General disorders and administration site conditions	2 (13.3)
Asthenia	1 (6.7)
Fatigue	1 (6.7)
Hepatobiliary disorders	1 (6.7)
Bile duct obstruction	1 (6.7)
Investigations	2 (13.3)
Alanine aminotransferase increased *	1 (6.7)
Aspartate aminotransferase increased *	1 (6.7)
Blood alkaline phosphatase increased *	1 (6.7)
Blood bilirubin increased *	1 (6.7)
Platelet count increased *	1 (6.7)
Metabolic and nutritional disorders	2 (13.3)
Dehydration	1 (6.7)
Failure to thrive	1 (6.7)
Respiratory, thoracic, and mediastinal disorders	1 (6.7)
Pneumonia	1 (6.7)

* “Increased” refers to values above the upper limit of normal (ULN). Abbreviations: MedDRA = Medical Dictionary for Regulatory Activities; TEAE = Treatment-Emergent Adverse Event; CTCAE = Common Terminology Criteria for Adverse Events.

## Data Availability

Data can be available upon reasonable request from Sunil Gupta (former employee of OncoQuest Pharmaceuticals, Inc.).
